# Proposed Modification of the pN2 Classification of the 8th Edition AJCC Staging System for Esophageal Squamous Cell Carcinoma: A Preliminary Study Based on the Chinese Population

**DOI:** 10.1155/2021/8871884

**Published:** 2021-03-09

**Authors:** Kexing Xi, Hui Yu

**Affiliations:** ^1^Department of Colorectal Surgery and State Key Lab of Molecular Oncology, National Cancer Center/National Clinical Research Center for Cancer/Cancer Hospital, Chinese Academy of Medical Sciences and Peking Union Medical College, Beijing 100021, China; ^2^Department of Thoracic Surgery, Sun Yat-sen University Cancer Center, Guangzhou 510060, China; ^3^State Key Laboratory of Oncology in South China, Collaborative Innovation Center for Cancer Medicine, Guangzhou 510060, China

## Abstract

**Objective:**

To evaluate the efficacy of the nodal staging of the 8th edition AJCC staging system for esophageal squamous cell carcinoma (ESCC) and propose a modification of the current pN2 classification.

**Methods:**

1188 patients who underwent esophagectomy for ESCC at Sun Yat-sen University Cancer Center in Guangzhou (Guangdong, China) between January 2005 and June 2010 were reviewed. We used the X-tile software to determine the optimal cutoff points. Kaplan–Meier method and log-rank test were used to compare the differences of survival. Multivariate Cox regression analysis was performed for the factors that were statistically significant in univariate analysis.

**Result:**

In multivariate Cox regression analysis, alcohol consumption, pT status, and pN status were independent prognostic factors for overall survival (OS) according to the current pN classifications. And the observed 5-year OS rates for groups pN0, pN1, pN2, pN3 were 66.7%, 45.0%, 31.5%, and 21.5%, respectively (*P*<0.001). Based on the above results, the current pN2 classification was further subdivided as pN2a [3 metastatic lymph nodes (LNs)] and pN2b (4−6 metastatic LNs) groups. The 5-year OS rates for groups pN0, pN1, pN2a, pN2b, and pN3 were 66.7%, 45.0%, 37.7%, 26.3% and 21.5%, respectively (*P*<0.001). The rate of 5-year disease-free survival (DFS) was 60.0% for patients with pN0, compared with 36.8%, 29.3%, 20.8%, and 14.3% for those with pN1, pN2a, pN2b, and pN3, respectively (*P*<0.001).The current pN2 classification should be subdivided as pN2a (3 metastatic LNs) and pN2b (4–6 metastatic LNs) groups. The modified pN2 classification could better discriminate the survival differences between patients with 3–6 metastatic LNs for ESCC in the Chinese population.

## 1. Introduction

Globally, esophagealcancer (EC) is the seventh most common diagnosed cancer and the sixth most frequent cause of cancer-related deaths [1, 2].The histological type of EC mainly consisted of squamous cellcarcinomaand adenocarcinoma [3]. In China, more than 90% of EC cases is squamous cell carcinoma [4, 5]. The prognosis of EC is still unsatisfactory and the 5-year overall survival (OS) rate is not more than 25% [6, 7].

Lymph node (LN) status is one of the most important factors for the prognosis of EC patients [8]. Tumor-node-metastasis (TNM) staging system is applied to estimate prognosis and guide treatment plan making in clinical practice [9]. The current nodal staging system is based on the number of metastatic LNs. According to the eighth edition of the American Joint Committee on Cancer (AJCC) staging manual, the pN categories of EC are classified as pN0 (0), pN1 (1-2), pN2 (3-6), and pN3 (≥7) based on the number of metastatic LNs [10]. The current nodal staging system defines patients with 3–6 metastatic LNs as pN2, which is too general. The previous studies have demonstrated that the number of metastatic LNs is significantly associated with prognosis of EC [11-13]. Thus, the current pN2 classification should be divided into more subgroups.

In this study, data were collected from a large cohort of Chinese patients in a high-volume institution. We aimed to provide a proposal to subdivide the current pN2 classification, which could provide more prognostic information for esophageal squamous cellcarcinoma (ESCC) patients in the Chinese population.

## 2. Patients and Methods

### 2.1. Patients Selection

Patients who underwent esophagectomy for ESCC at Sun Yat-sen University Cancer Center in Guangzhou (Guangdong, China) between January 2005 and June 2010 were reviewed. This study was approved by the Ethics Committee of Sun Yat-sen University Cancer Center.

**Figure 1:**
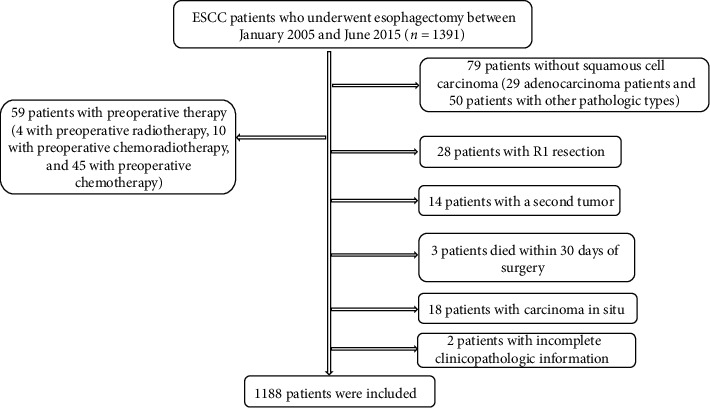
Selection flow of 1188 ESCC patients.

Patients were included based on the following eligibility criterion: patients who underwent radical esophagectomy with pathologically confirmed ESCC. Patients were not eligible according to the following criteria: (1) diagnosed with not squamous cell carcinoma; (2) patients with preoperative therapy, including radiotherapy, chemotherapy, or chemoradiotherapy; (3) patients who underwent R1/R2 resection; (4) patients with a second tumor; (5) those who died within 30 days of surgery; (6) patients with carcinoma in situ; (7) patients with incomplete clinicopathologic information; and (8) patients with distant metastasis. Finally, 1188 patients were included in the present study (Figure 1).

### 2.2. Follow-Up

All patients were followed up after surgery every 3 months for the first year, every 6 months for the next 2 years, and annually thereafter. The follow-up examinations including thoracoabdominal CT, endoscopy, tumor markers, and barium esophagography. The last follow-up date was conducted at July 1, 2015. The interval between the date of surgery and the date of death or the last follow-up was defined as overall survival (OS) time. The disease-free survival (DFS) was defined as the time period from surgery to the appearance of tumor progression or the date of cancer-related death.

### 2.3. Statistical Analysis

Statistical analyses were performed using SPSS 25.0 software (SPSS Inc., Chicago, IL, USA). The optimal cutoff points were identified using the X-tile software (version 3.6.1, Copyright Yale University, 2003) [14]. X-tile software determined the best cutoff points by using the minimum *p* value. Kaplan–Meier method and log-rank test were used to compare the differences of survival. Multivariate Cox regression analysis was performed for the factors that were statistically significant in univariate analysis. A two-tailed *P* value *<*0.05 was considered statistically significant.

## 3. Results

A total of 1188 patients with ESCC were enrolled in this study, including 928 male patients and 260 female patients. The mean and median age of all patients were 58.4 and 58.0 years, respectively (range, 30−88 years). 616 (51.9%) patients without metastatic LN and 572 (48.1%) had LN metastasis (Table 1). The number of N0, N1, N2, and N3 patients was 616 (51.9%), 319 (26.9%), 204 (17.2%), and 49 (4.1%), respectively.

**Figure 2:**
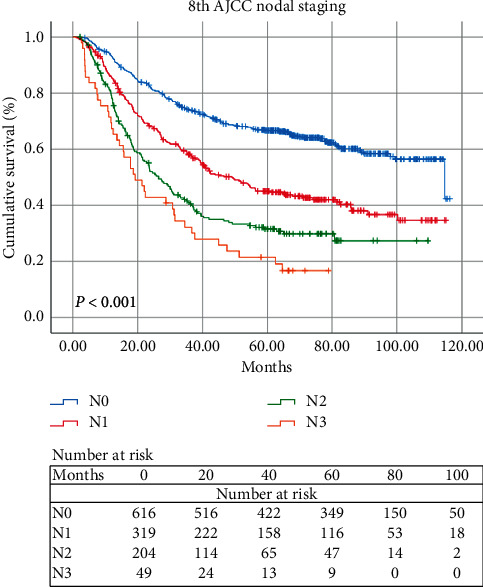
Kaplan–Meier survival curves for OS of 1188 ESCC patients based on the 8th edition AJCC nodal staging.

Cox univariate and multivariate analyses were used to evaluate the prognostic factors of OS based on the current pN classifications. Univariate analysis demonstrated that smoking status, alcohol consumption, tumor length, pT status, pN status, and differentiation were independent prognostic factors of OS. In multivariate analysis, only alcohol consumption, tumor length, pT status, and pN status were independent prognostic factors (Table 1). Kaplan–Meier curves were applied to compare the survival rates among different pN groups. The analysis results demonstrated that the observed 5-year OS rates for groups pN0, pN1, pN2, and pN3 were 66.7%, 45.0%, 31.5%, and 21.5%, respectively (*P*<0.001, Figure 2). The 5-year OS rate was significantly worse with the increasing pN classifications.

**Figure 3:**
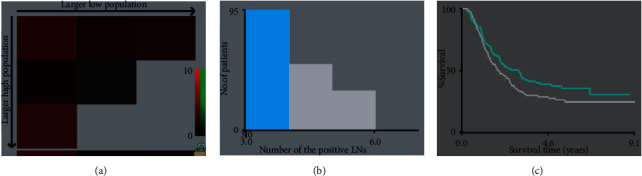
X-tile analysis for the optimal cutoff points.

**Figure 4:**
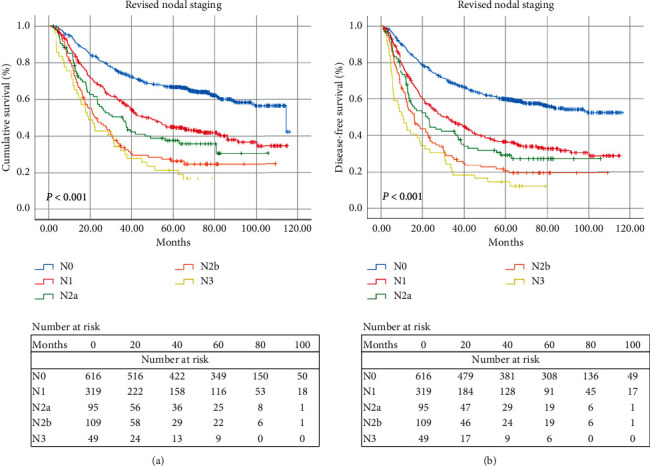
(a) Kaplan–Meier survival curves for OS of 1188 ESCC patients based on the revised nodal staging. (b) Kaplan–Meier survival curves for DFS of 1188 ESCC patients based on the revised nodal staging.

Based on the above results, the current pN2 classification was further subdivided as pN2a (3 metastatic LNs) and pN2b (4-6 metastatic LNs) groups by using the X-tile software (Figure 3). Based on the revised pN classifications, univariate and multivariate analyses were performed to identify the risk factors of OS and DFS. In univariate analysis, smoking status, alcohol consumption, tumor length, pT status, revised pN status, and differentiation were associated with OS and DFS significantly. However, only alcohol consumption, tumor length, pT status, and revised pN status were independent prognostic factors of OS in multivariate analysis (Table 2). Moreover, only tumor length, pT status, and revised pN status were significantly associated with DFS in multivariate analysis (Table 3).The 5-year OS and DFS rates based on the revised pN classifications were shown in Figures 4(a) and 4(b). The 5-year OS rates for groups pN0, pN1, pN2a, pN2b, and pN3 were 66.7%, 45.0%, 37.7%, 26.3%, and 21.5%, respectively (*P*<0.001). The rate of 5-year DFS was 60.0% for patients with pN0, compared with 36.8%, 29.3%, 20.8%, and 14.3% for those with pN1, pN2a, pN2b, and pN3, respectively (*P*<0.001). Increased pN classifications demonstrated significantly decreased survival.

## 4. Discussion

LN status is an important prognostic factor for EC patients. In clinical practice, the 8th edition AJCC classification system is widely applied in staging of EC. The 8th nodal staging defines 3–6 metastatic LNs as pN2 in EC, which is too general and could not provide more detail of the survival information.

In the present study, we validated the current pN classifications of the 8th edition AJCC classification system in the first stage and found that there were significant differences in survival between different pN groups. After that, the current pN2 classification was subdivided as pN2a and pN2b groups. What was more, patients with pN2a had a better survival than those with pN2b significantly and significant survival differences existed between each revised pN classification. The results of our study showed that the revised pN2 classifications could provide more accurate prognosis information for EC patients.

As a reasonable nodal classification system, there should be a higher predictive value for prognosis and a different survival rate between each group. Although the current nodal staging provided a more exact evaluation of survival than the sixth edition, it had not the ability to distinguish the survival of EC patients with 3–6 metastatic LNs [15]. However, the revised pN2 classification showed a good discriminatory ability for survival in these patients. The current pN2 classification of EC did not consider the effect of the specified number of metastatic LNs, which might not be perfect.

Previous studies demonstrated that a nodal staging system based on the number of metastatic LNs may be more sensitive for the survival estimation for EC than classification based on the location of LNs [16-18]. Compared with other indicators such as LN ratio (LNR, the ratio between the number of metastatic LNs and the total number of examined LNs) and log odds ratio (LODDS, the log of the ratio between the number of positive LNs and the number of negative LNs), the indicator—the number of metastatic LNs—was simpler and thus easy to generalize. Thus, our proposal to subdivide the pN2 classification was still based on the number of metastatic LNs.

In univariate and multivariate analyses, we also found that alcohol consumption was an independent prognostic factor of survival. This was consistent with previous studies [19, 20]. However, no significant survival differences were observed between the patients with <15 LNs examined and those with ≥15 LNs examined (*P*=0.073). This might be because of the sample size of this study and the bias of retrospective study. The *P* value (0.073) was closed to 0.05 and we thought that, with the sample size increased, there would be significant survival differences between these two groups.

It is well known that the clinicopathological features of EC vary widely between patients in Asian and Western countries [21]. Squamous cell carcinoma is the most common pathological type in China, and it is considered that more data from Chinese patients are essential to validate the generalizability of the 8th edition AJCC nodal staging system for ESCC [5]. A relatively large sample size solely from Chinese ESCC patients in one institution was included in this study, which could help to decrease the heterogeneous characteristics. Additionally, we determined the optimal cutoff values using the X-tile, which was one of the most convenient and objective methods [14, 22]. As such, the results from our data were more precise and significant, which could better represent the prognostic characteristics of ESCC in the Chinese population.

Some limitations in the present study should be acknowledged. Firstly, the bias of a retrospective investigation was unavoidable. Secondly, the number of pN3 cases was small, which implied that the results needed further validation. This was a preliminary study based on single-center and we planned to enroll other databases to verify the conclusion and further subgroup the pN2 classification next.

In conclusion, we proposed a modification of the current pN2 classification for ESCC. The modified pN2 classification could better discriminate the survival differences between patients with 3–6 metastatic LNs for ESCC in the Chinese population.

## Figures and Tables

**Table 1:** Univariate and multivariate Cox regression analyses of prognostic factors for OS in 1188 ESCC patients based on the 8th edition AJCC nodal staging.

Variable	*N* (%)	Univariate analysis		Multivariate analysis	
		HR (95% CI)	*P*	HR (95% CI)	*P*
Gender			0.481		
Male	928 (78.1)	Reference			
Female	260 (21.9)	0.932 (0.765−1.134)			
Age (years)			0.063		
≤65	935 (78.7)	Reference			
＞65	253 (21.3)	1.199 (0.990−1.452)			
Smoking status			0.025		
Never	420 (35.4)	Reference			
Former	768 (64.6)	1.219 (1.025−1.450)			
Alcohol consumption			<0.001		0.005
No	924 (77.8)	Reference		Reference	
Yes	264 (22.2)	1.431 (1.192−1.718)		1.301 (1.082−1.564)	
Tumor length (cm)			<0.001		0.043
≤3	317 (26.7)	Reference		Reference	
3−5	364 (30.6)	1.217 (0.972−1.523)	0.086	0.796 (0.626−1.013)	0.064
≥5	507 (42.7)	1.523 (1.242−1.869)	<0.001	1.001 (0.801−1.251)	0.992
Tumor location			0.282		
Upper	117 (9.8)	Reference			
Middle	761 (64.1)	1.128 (0.851−1.497)	0.402		
Lower	310 (26.1)	0.974 (0.712−1.332)	0.869		
pT status			<0.001		<0.001
T1	119 (10.0)	Reference		Reference	
T2	232 (19.5)	2.212 (1.456−3.358)	<0.001	1.937 (1.260−2.979)	0.003
T3	796 (67.0)	2.968 (2.024−4.352)	<0.001	2.381 (1.575−3.599)	<0.001
T4	41 (3.5)	6.071 (3.608−10.215)	<0.001	4.296 (2.474−7.459)	<0.001
pN status			<0.001		<0.001
N0	616 (51.9)	Reference		Reference	
N1	319 (26.9)	1.880 (1.545−2.288)	<0.001	1.742 (1.428−2.124)	<0.001
N2	204 (17.2)	2.861 (2.309−3.546)	<0.001	2.514 (2.019−3.130)	<0.001
N3	49 (4.1)	4.051 (2.889−5.681)	<0.001	3.683 (2.616−5.184)	<0.001
Differentiation			0.024		
Well	253 (21.3)	Reference			
Moderate	587 (49.4)	1.155 (0.928−1.438)	0.195		
Poor	347 (29.2)	1.374 (1.086−1.737)	0.008		
Surgical approach			0.146		
Left thoracotomy	762 (64.1)	Reference			
Right thoracotomy	426 (35.9)	0.881 (0.742−1.045)			
Anastomosis			0.659		
Hand-sewn	168 (14.1)	Reference			
Stapled	1020 (85.9)	0.950 (0.755−1.194)			
Adjuvant therapy			0.469		
No	987 (83.1)	Reference			
Yes	201 (16.9)	1.083 (0.872−1.345)			
Number of resected LNs			0.073		
<15	357 (30.1)	Reference			
≥15	831 (69.9)	0.854 (0.719−1.015)			

HR: hazard ratio, CI: confidence interval, LNs: lymph nodes, and OS: overall survival.

**Table 2:** Univariate and multivariate Cox regression analyses of prognostic factors for OS in 1188 ESCC patients based on the 8th edition AJCC nodal staging.

Variable	*N* (%)	Univariate analysis		Multivariate analysis	
		HR (95% CI)	*P*	HR (95% CI)	*P*
Gender			0.481		
Male	928 (78.1)	Reference			
Female	260 (21.9)	0.932 (0.765−1.134)			
Age (years)			0.063		
≤65	935 (78.7)	Reference			
>65	253 (21.3)	1.199 (0.990−1.452)			
Smoking status			0.025		
Never	420 (35.4)	Reference			
Former	768 (64.6)	1.219 (1.025−1.450)			
Alcohol consumption			<0.001		0.007
No	924 (77.8)	Reference		Reference	
Yes	264 (22.2)	1.431 (1.192−1.718)		1.289 (1.072−1.550)	
Tumor length (cm)			<0.001		0.036
≤3	317 (26.7)	Reference		Reference	
3−5	364 (30.6)	1.217 (0.972−1.523)	0.086	0.800 (0.628−1.017)	0.069
≥5	507 (42.7)	1.523 (1.242−1.869)	<0.001	1.017 (0.813−1.271)	0.886
Tumor location			0.282		
Upper	117 (9.8)	Reference			
Middle	761 (64.1)	1.128 (0.851−1.497)	0.402		
Lower	310 (26.1)	0.974 (0.712−1.332)	0.869		
pT status			<0.001		<0.001
T1	119 (10.0)	Reference		Reference	
T2	232 (19.5)	2.212 (1.456−3.358)	<0.001	1.944 (1.264−2.989)	0.002
T3	796 (67.0)	2.968 (2.024−4.352)	<0.001	2.368 (1.567−3.580)	<0.001
T4	41 (3.5)	6.071 (3.608−10.215)	<0.001	4.425 (2.547−7.689)	<0.001
pN status			<0.001		<0.001
N0	616 (51.9)	Reference		Reference	
N1	319 (26.9)	1.881 (1.546−2.289)	<0.001	1.742 (1.429−2.125)	<0.001
N2a	95 (8.0)	2.445 (1.831−3.266)	<0.001	2.104 (1.567−2.826)	<0.001
N2b	109 (9.2)	3.280 (2.531−4.251)	<0.001	2.936 (2.256−3.821)	<0.001
N3	49 (4.1)	4.054 (2.891−5.685)	<0.001	3.688 (2.620−5.191)	<0.001
Differentiation			0.024		
Well	253 (21.3)	Reference			
Moderate	587 (49.4)	1.155 (0.928−1.438)	0.195		
Poor	347 (29.2)	1.374 (1.086−1.737)	0.008		
Surgical approach			0.146		
Left thoracotomy	762 (64.1)	Reference			
Right thoracotomy	426 (35.9)	0.881 (0.742−1.045)			
Anastomosis			0.659		
Hand-sewn	168 (14.1)	Reference			
Stapled	1020 (85.9)	0.950 (0.755−1.194)			
Adjuvant therapy			0.469		
No	987 (83.1)	Reference			
Yes	201 (16.9)	1.083 (0.872−1.345)			
Number of resected LNs			0.073		
<15	357 (30.1)	Reference			
≥15	831 (69.9)	0.854 (0.719−1.015)			

HR: hazard ratio, CI: confidence interval, LNs: lymph nodes, and OS: overall survival.

**Table 3:** Univariate and multivariate Cox regression analyses of prognostic factors for DFS in 1188 ESCC patients based on the revised pN classifications.

Variable	*N* (%)	Univariate analysis		Multivariate analysis	
		HR (95% CI)	*P*	HR (95% CI)	*P*
Gender			0.319		
Male	928 (78.1)	Reference			
Female	260 (21.9)	0.909 (0.755−1.096)			
Age (years)			0.596		
≤65	935 (78.7)	Reference			
＞65	253 (21.3)	1.051 (0.874−1.265)			
Smoking status			0.048		
Never	420 (35.4)	Reference			
Former	768 (64.6)	1.178 (1.001−1.386)			
Alcohol consumption			0.001		
No	924 (77.8)	Reference			
Yes	264 (22.2)	1.334 (1.119−1.591)			
Tumor length (cm)			<0.001		0.008
≤3	317 (26.7)	Reference		Reference	
3−5	364 (30.6)	1.142 (0.926−1.408)	0.215	0.795 (0.635−0.997)	0.047
≥5	507 (42.7)	1.468 (1.213−1.778)	<0.001	1.052 (0.853−1.297)	0.635
Tumor location			0.300		
Upper	117 (9.8)	Reference			
Middle	761 (64.1)	1.004 (0.775−1.301)	0.978		
Lower	310 (26.1)	0.870 (0.652−1.160)	0.343		
pT status			<0.001		<0.001
T1	119 (10.0)	Reference		Reference	
T2	232 (19.5)	1.995 (1.387−2.869)	<0.001	1.761 (1.208−2.566)	0.003
T3	796 (67.0)	2.416 (1.735−3.363)	<0.001	1.916 (1.334−2.751)	<0.001
T4	41 (3.5)	4.495 (2.783−7.259)	<0.001	3.210 (1.930−5.338)	<0.001
pN status			<0.001		<0.001
N0	616 (51.9)	Reference		Reference	
N1	319 (26.9)	1.957 (1.628−2.353)	<0.001	1.877 (1.558−2.261)	<0.001
N2a	95 (8.0)	2.431 (1.850−3.193)	<0.001	2.140 (1.621−2.826)	<0.001
N2b	109 (9.2)	3.177 (2.483−4.066)	<0.001	3.020 (2.351−3.878)	<0.001
N3	49 (4.1)	4.353 (3.147−6.021)	<0.001	4.088 (2.941−5.682)	<0.001
Differentiation			0.015		
Well	253 (21.3)	Reference			
Moderate	587 (49.4)	1.157 (0.942−1.421)	0.165		
Poor	347 (29.2)	1.373 (1.101−1.713)	0.005		
Surgical approach			0.348		
Left thoracotomy	762 (64.1)	Reference			
Right thoracotomy	426 (35.9)	0.926 (0.788−1.088)			
Anastomosis			0.676		
Handsewn	168 (14.1)	Reference			
Stapled	1020 (85.9)	0.954 (0.767−1.188)			
Adjuvant therapy			0.129		
No	987 (83.1)	Reference			
Yes	201 (16.9)	1.168 (0.956−1.428)			
Number of resected LNs			0.197		
<15	357 (30.1)	Reference			
≥15	831 (69.9)	0.898 (0.762−1.058)			

HR: hazard ratio, CI: confidence interval, LNs: lymph nodes, and DFS: disease-free survival.

## Data Availability

We may balance the potential benefits and risks for each request and then provide the data that could be shared.
